# Supplementary material for: Association between oral microbiome and seven types of cancers in East Asian population: a two-sample Mendelian randomization analysis
Source: Front Mol Biosci. 2023 Nov 21;10:1327893. doi: 10.3389/fmolb.2023.1327893 (PMC10702768; doi:10.3389/fmolb.2023.1327893)
Supplement: Supplementary file 1 [file DataSheet1.ZIP › Supplementary Materials/MR plots for tongue/tongue═╝/Gastric cancer/pheno.2687_to_gastric cancer_leave_one_out.pdf]

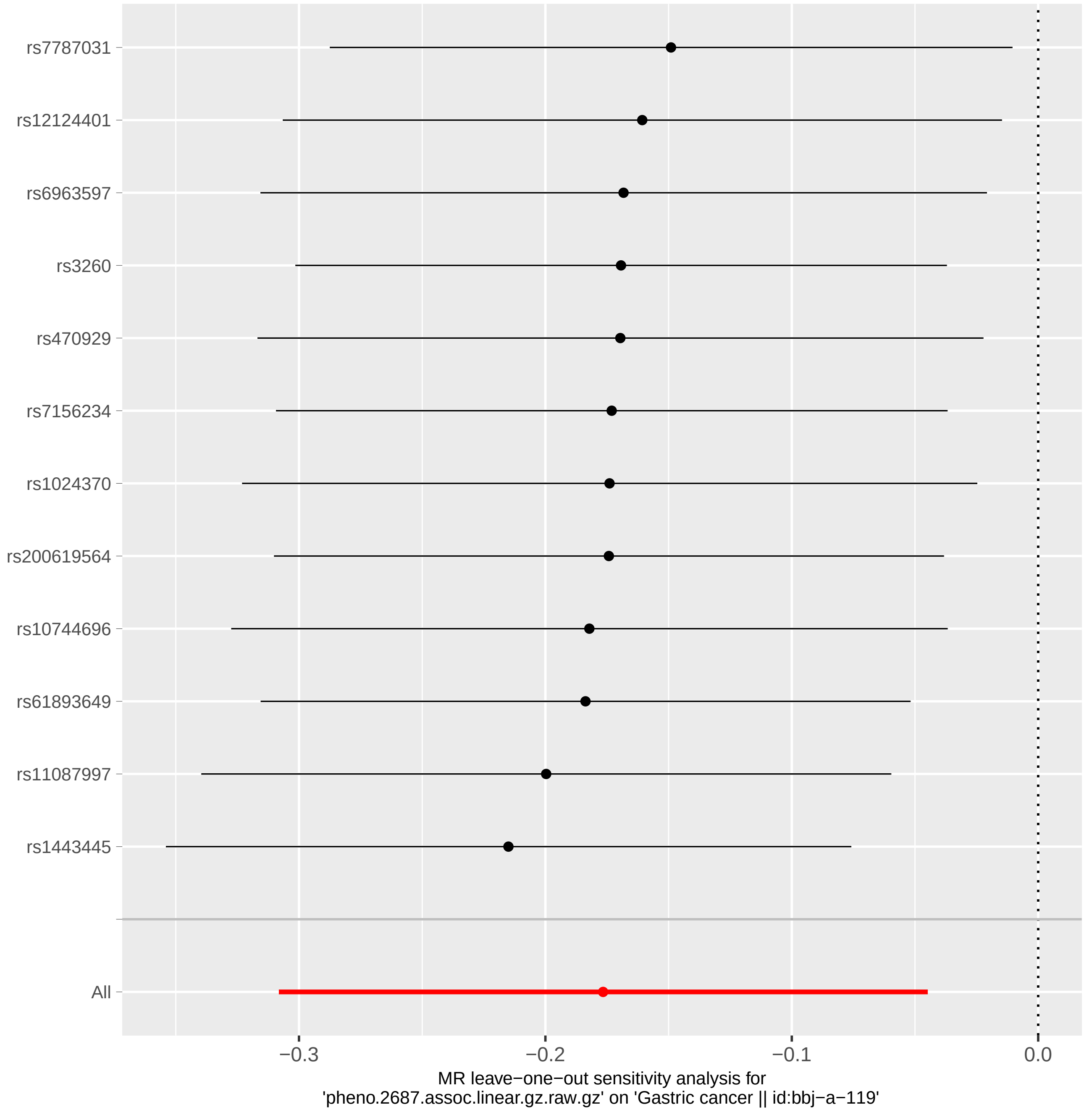
rs7787031
rs12124401
rs6963597
rs3260
rs470929
rs7156234
rs1024370
rs200619564
rs10744696
rs61893649
rs11087997
rs1443445
All
−0.3
−0.2
−0.1
0.0
MR leave−one−out sensitivity analysis for
'pheno.2687.assoc.linear.gz.raw.gz' on 'Gastric cancer || id:bbj−a−119'
